# COMPARATIVE ANALYSIS OF TREATMENTS FOR FOREARM FRACTURES IN CHILDREN: A SYSTEMATIC REVIEW AND META-ANALYSIS

**DOI:** 10.1590/1413-785220253306e290231

**Published:** 2025-11-10

**Authors:** Airton Pereira da Costa, Erika Tonarelli Rodrigues, Hassan Ahmad Hauache, Mariana Ayumi Fujisaki, Eiffel Tsuyoshi Dobashi

**Affiliations:** 1 Universidade Federal de Sao Paulo, Escola Paulista de Medicina, Departamento Ortopedia e Traumatologia, Sao Paulo, SP, Brazil.

**Keywords:** Fractures, bone, Forearm, Child, Orthopedic Procedures, Therapeutics, Postoperative Complications, Fraturas Ósseas, Antebraço, Criança, Procedimentos Ortopédicos, Terapêutica, Complicações Pós-Operatórias

## Abstract

To compare the clinical outcomes of children with forearm bone fractures undergoing surgical treatment with intramedullary fixation with TEN rods and Kirschner wires. A systematic review of the literature was carried out, conducting a search for data in the Pubmed/Medline, Science Direct and Scielo databases. The quality of the trials was assessed by the MINORS tool and the meta-analysis of the studies was performed using the R software (version 4.4.0). 16 studies were selected, representing 1,075 patients, with a predominance of males, where the mean age range varied from 8.32 to 14.2 years. Applying the MINORS Scale, the quality of the studies was good (≥ 11). The meta-analysis of the studies revealed a statistically significant increase in the risk of adverse events in the experimental group compared to the control group, with a risk ratio (RR) of 1.35 (95% CI: 1.03 to 1.76). The combined mean difference (raw mean) between the experimental group and the control group was −12.42 minutes (95% CI: −13.75 to −11.10) in the fixed-effect model, indicating a significant reduction in surgical time for the experimental group. In the random-effect model, the mean difference was −21.62 minutes (95% CI: −33.30 to −9.94). Regarding fracture consolidation time, the fixed-effect model indicated a raw mean difference of 0.99 (95% CI: 0.61 to 1.36). Furthermore, heterogeneity was moderate to high, with an I² of 73% (p < 0.01). Intramedullary fixation with TEN nails and Kirschner wires presents a diversity of clinical outcomes and complications. The systematic review highlighted the importance of choosing the appropriate treatment method, considering the patient characteristics and the nature of the fracture. ***Level of Evidence II; Systematic Review***.

## RESUMO

Comparar os desfechos clínicos de crianças com fraturas dos ossos do antebraço submetidas ao tratamento cirúrgico fixação intramedular com hastes de TEN e fios de Kirschner. Realizou-se uma revisão sistemática da literatura, conduzindo a busca de dados nas bases Pubmed/Medline, Science Direct e Scielo. A qualidade dos ensaios foi avaliada pela ferramenta MINORS e a meta-análise dos estudos foi realizada utilizando o software R (versão 4.4.0). 16 estudos foram selecionados, representando 1.075 pacientes, com predominância do sexo masculino, onde faixa etária média variou de 8,32 a 14,2 anos. Aplicando a Escala MINORS, obteve-se que a qualidade dos estudos foi considerada boa (≥ 11). A meta-análise dos estudos revelou em relação aos efeitos adversos o aumento estatisticamente significativo no risco de eventos adversos no grupo experimental comparado ao controle, com uma razão de risco (RR) de 1,35 (IC 95%: 1,03 a 1,76). O tempo de cirurgia demonstrou que a diferença de média combinada (média crua) entre o grupo experimental e o grupo controle foi de −12,42 minutos (IC 95%: −13,75 a −11,10) no modelo de efeito fixo, indicando uma redução significativa no tempo de cirurgia para o grupo experimental. Já no modelo de efeito aleatório, a diferença de média foi de −21,62 minutos (IC 95%: −33,30 a −9,94). No tempo de consolidação da fratura verificou-se que o modelo de efeito fixo indicou uma diferença de média crua de 0,99 (IC 95%: 0,61 a 1,36). Além disso, a heterogeneidade foi moderada a alta, com um I² de 73% (p < 0,01). A fixação intramedular com haste TEN e fios de Kirschner, apresenta diferentes desfechos clínicos, incluindo complicações diversas. A revisão sistemática destacou a importância da escolha adequada do método de tratamento, considerando as características do paciente e a natureza da fratura. ***Nível de Evidência II; Revisão Sistemática***.

## INTRODUCTION

Diaphyseal forearm fractures are frequent in children and adolescents, representing 74% of all immature skeletal injuries of the upper limb.^1^ This type of trauma encompasses a variety of injury patterns, including isolated radial shaft fractures, isolated ulnar shaft fractures, fractures of both forearm bones, as well as Galeazzi and Monteggia fracture-dislocations.^2,3^ Although less common than distal radius fractures, diaphyseal forearm fractures still represent a significant challenge for orthopedic surgeons.^3-5^ While the distal forearm is the most common fracture site, standardized treatment and follow-up protocols for these injuries are not yet established.^6,7^

While the distal forearm is the most common fracture site, standardized treatment and follow-up protocols for these injuries are not yet established.^8^

In the management of pediatric forearm fractures, closed reduction followed by cast immobilization is considered the gold standard.^4,7^ However, there has been a growing trend toward surgical stabilization of diaphyseal fractures. Overall, the evidence suggests that surgery should be reserved for cases in which satisfactory alignment cannot be achieved through closed reductions.^9^ In certain pediatric fractures, the choice between conservative and surgical treatment has been influenced by several factors, including technological advances, the availability of imaging equipment in operating rooms, safer anesthesia, improved implants specifically designed for the pediatric skeleton, and the surgical training of orthopedic surgeons in minimally invasive techniques.^9^

It is noteworthy that several approaches for treating forearm bone fractures, including intramedullary fixation (IM) using Kirschner wires (K-wires) or Titanium Elastic Nails (TEN rods), have emerged as predominant methods for displaced and unstable diaphyseal forearm injuries in children.^10,11^ Nonetheless, it is well recognized that not all patients are suitable candidates for closed manual reduction followed by intramedullary fixation.^12^

Therefore, considering the diversity of aspects related to this topic in the pediatric population, it is essential to deepen the understanding of the best available treatment options and to determine their clinical effectiveness.^13^ Given the lack of consensus regarding optimal treatment and follow-up protocols for these injuries, we consider it pertinent to conduct a comparative analysis between the most common therapeutic options. This investigation aims to improve clinical practice and provide a scientifically sound basis for decision-making, thereby optimizing clinical and functional outcomes for patients with forearm fractures.

In this context, the present study aims primarily to compare the clinical outcomes of children with forearm fractures who underwent surgical treatment using TEN rods and Kirschner wires.

## MATERIALS AND METHODS

This study presents a systematic review conducted in accordance with the protocol established by the Preferred Reporting Items for Systematic Reviews and Meta-Analyses (PRISMA).^14^

Primary studies were included, such as cross-sectional research, cohort studies, randomized clinical trials, and case reports, which addressed treatments for forearm fractures in the pediatric population. No language restrictions were applied, and studies published in the last five years were considered. Review studies and duplicates were excluded.

The guiding question was structured according to the PICO approach, which includes the following elements: the studied population (P), the intervention performed (I), the comparison made (C), and the outcome assessed (O). The population consisted of pediatric patients with forearm fractures; the interventions included treatments with plaster, intramedullary fixation with TEN rods, or Kirschner wires, compared with various treatment types; and the outcomes included treatment effectiveness, associated complications, recovery time, and post-treatment functionality. Based on this strategy, the following research question was formulated: "What is the effectiveness and what are the associated complications of different treatments for forearm fractures in children, comparing plaster, intramedullary TEN rods, and Kirschner wires?"

Searches were conducted from June to July 2024. The databases used were: Medical Literature Analysis and Retrieval System Online/National Library of Medicine (MEDLINE®/PubMed®), Science Direct, and Scientific Electronic Library Online (Scielo). Additional searches were carried out in the bibliographies of the selected studies to improve coverage and incorporate studies not initially identified. In the PubMed database, filters for the last 5 years and full-text articles were applied. No filters were applied in Scielo.

The descriptors were selected from the Health Sciences Descriptors/Medical Subject Headings (DeCS/MeSH) in Portuguese and English, combined using the Boolean operators AND and OR: "fratura do antebraço em crianças," "hastes elásticas de titânio," "fios de Kirschner," "gesso" OR "forearm fracture in children," "titanium elastic rods," "Kirschner wires," "plaster."

Two researchers independently evaluated all included studies. Potentially relevant articles were examined in full. Divergences were discussed among the reviewers and, when necessary, submitted to a third evaluator.

**Figure 1:**
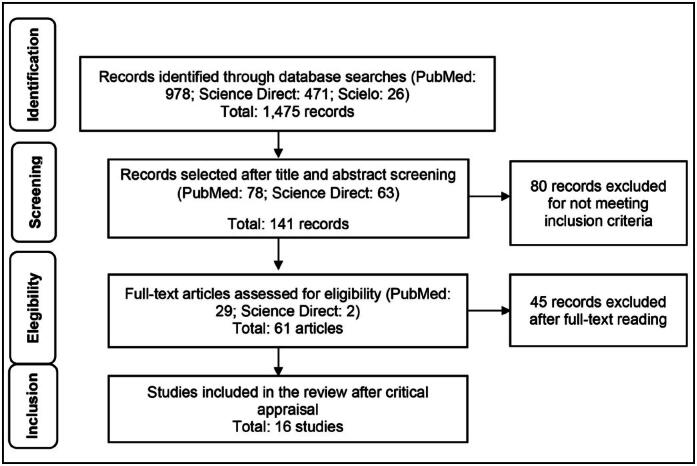
Schematic representation of the methods of identification, screening, eligibility, and inclusion of studies in the review, adapted according to the PRISMA Flow Diagram.

The assessment of the studies was performed by two independent evaluators. The quality of the trials was assessed using the MINORS tool^15^ for observational studies. Screening involved analysis of article titles and abstracts, followed by full-text reading of those deemed relevant (Figure 1). During the search process, data were meticulously recorded in a spreadsheet and organized into tables to facilitate analysis.

**Figure 2:**
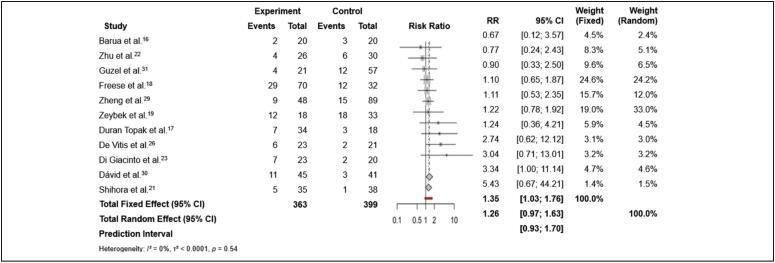
Meta-analysis of the binary outcome (adverse events) in patients treated with TEN rods and/or Kirschner wires versus the control group (other treatments).

Meta-analysis of the studies was conducted using R software (version 4.4.0) with the meta package. A total of five meta-analyses were carried out. The first analysis involved binary outcomes (occurrence or non-occurrence of complications in the treatment and control groups) in order to evaluate adverse events in patients. (Figure 2)

**Figure 3:**
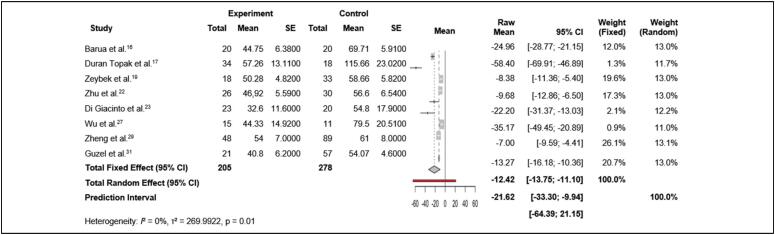
Meta-analysis of continuous outcome (surgical time) in patients treated with TEN rods and/or Kirschner wires versus the control group.

In addition, three meta-analyses with continuous variables (meta-analysis of continuous outcome data) were conducted, using the random-effects model. This model allowed us to calculate an overall mean across all studies that reported the mean and standard error of continuous variables, such as surgical time, length of hospital stay, and bone consolidation time, in both the experimental and control groups. The results of the meta-analysis on surgical time are presented in Figure 3.

It should be noted that the meta-analysis of hospital stay duration included only a few studies, since it was necessary to exclude those that did not provide data to establish a control group. To address this limitation, an additional meta-analysis was conducted considering hospital stay across all studies, regardless of whether results were reported for the control group. In this case, the procedure followed was a meta-analysis of single means, also using the random-effects model.

## RESULTS

A total of 1,475 articles were initially identified in the search. The summary of the article selection process is presented in Figure 1. After evaluation of titles and abstracts, followed by the selection and detailed analysis of the articles, 16 studies were deemed eligible to compose this systematic review.

The systematic review followed the PRISMA recommendations, illustrated in Figure 1.

The studies included in this systematic review consisted of observational research investigating different treatments for forearm fractures, with emphasis on intramedullary (IM) fixation using Kirschner wires (K-wires) or Titanium Elastic Nails (TEN rods).

The overall sample comprised approximately 1,075 patients, with a predominance of male participants across all studies. The mean age range of participants varied from 8.32 to 14.2 years, with a greater proportion of studies focused on children. The methodological characteristics of the selected studies are detailed in Table 1.

**Table 1:** Summary of demographic data and treatments across all included studies.

Author/Year	Study Type	Intervention Groups	Sex (M/F)	Age (years)	Follow-up (months)
Barua et al.^16^	Retrospective	▪ PO: 20 ▪ TEN: 20	▪ PO: 14/6 ▪ TEN: 14/6	▪ PO: 10.95 ± 2.35 ▪ TEN: 10.40 ± 2.41	▪ PO: NR ▪ TEN: NR
Duran Topak et al.^17^	Retrospective	▪ PO: 18 ▪ TEN: 34	▪ PO: 17/1 ▪ TEN: 28/6	▪ PO: 13.66 ± 1.45 ▪ TEN: 11.73 ± 1.60	▪ PO: 29.55 ▪ TEN: 30.85
Freese et al.^18^	Retrospective	▪ TENK: 70 ▪ ORIF: 32	▪ TENK: 44/26 ▪ ORIF: 22/10	▪ TENK: 12.1 ▪ ORIF: 14.2	▪ TENK: 6 ▪ ORIF: 3.3
Zeybek, Akti et al.^19^	Retrospective	▪ FPP: 19 ▪ TEN: 18 ▪ FH: 14	▪ FPP: 8/11 ▪ TEN: 8/10 ▪ FH: 5/9	▪ FPP: 11.00 ± 2.26 ▪ TEN: 10.11 ± 2.37 ▪ FH: 8.57 ± 2.24	▪ FPP: 6 ▪ TEN: 6 ▪ FH: 6
Soudy et al.^20^	Prospective	▪ TEN: 18	▪ TEN: 13/5	▪ TEN: 8.88	▪ TEN: 6
Shihora et al.^21^	Cross-sectional	▪ TEN: 35 ▪ CO: 38	▪ TEN + CO: 40/33	▪ TEN + CO: 8.32	▪ TEN + CO: 6
Zhu et al.^22^	Prospective	▪ FPD: 30 ▪ FH: 26	▪ FPD: 15/15 ▪ FH: 15/11	▪ FPD: 13.33 ± 1.54 ▪ FH: 13.27 ± 1.64	▪ FPD: 8 ▪ FH: 8
Di Giacinto et al.^23^	Retrospective	▪ FK: 23 ▪ ORIF: 20	▪ FK: 15/8 ▪ ORIF: 13/7	▪ FK: 12.86 ± 0.64 ▪ ORIF: 13.02 ± 1.77	▪ FK: 16.86 ▪ ORIF: 16.37
Jain et al.^24^	Retrospective	▪ TEN: 65	▪ TEN: 40/25	▪ TEN: 9.13	▪ TEN: 5.84
Pogorelić et al.^25^	Retrospective	▪ TEN: 173	▪ TEN: 126/47	▪ TEN: 11.0	▪ TEN: 68
De Vitis et al.^26^	Retrospective	▪ FES: 21 ▪ FK: 23	▪ FES: 16/5 ▪ FK: 18/5	▪ FES: 8.4 ± 1.6 ▪ FK: 8.5 ± 1.7	▪ FES: 3.4 ▪ FK: 2.4
Wu et al.^27^	Case-control	▪ TEN: 15 ▪ FK: 11	▪ TEN: 10/5 ▪ FK: 9/2	▪ TEN: 7.7 ± 2.0 ▪ FK: 6.4 ± 1.6	▪ TEN: 14 ▪ FK: 14
Acharya et al.^28^	Retrospective	▪ IM: 31	▪ IM: 22/9	▪ IM: 12.90	▪ IM: 8.51
Zheng et al.^29^	Retrospective	▪ ESIN: 48 ▪ FPD: 44 ▪ Hybrid: 45	▪ ESIN: 30/18 ▪ FPD: 25/18 ▪ Hybrid: 28/17	▪ ESIN: 13.5 ± 1.9 ▪ FPD: 13.4 ± 1.9 ▪ Hybrid: 13.2 ± 2.1	▪ ESIN: 14.8 ▪ FPD: 14.9 ▪ Hybrid: 15.0
Dávid et al.^30^	Retrospective	▪ ESIN: 45 ▪ RESIN: 41	▪ ESIN: 29/16 ▪ RESIN: 31/10	▪ ESIN: 10.4 ▪ RESIN: 8.4	▪ ESIN: NR ▪ RESIN: NR
Guzel et al.^31^	Retrospective	▪ TEN: 21 ▪ HF: 19 ▪ I-KW: 20 ▪ FPD: 18	▪ TEN: 11/10 ▪ HF: 11/8 ▪ I-KW: 9/11 ▪ FPD: 10/8	▪ TEN: 10.8 ± 2.2 ▪ HF: 11.5 ± 2.1 ▪ I-KW: 10.9 ± 2.1 ▪ FPD: 12.1 ± 1.9	▪ TEN: 12 ▪ HF: 12 ▪ I-KW: 12 ▪ FPD: 12

Legend: PO: plate osteosynthesis (plating); TEN: Titanium Elastic Nail (TEN rods); TENK: TEN rods + Kirschner wires; ORIF: open reduction and internal fixation with plate and screws; NR: not reported; FPP: plate–screw fixation; FH: hybrid fixation using elastic intramedullary fixation + plate–screw fixation; CO: conservative treatment with splint and cast; FPD: double-plate fixation (dual plating); FK: intramedullary fixation with Kirschner wires (K-wires); FES: Epibloc system fixation; IM: flexible intramedullary rod; ESIN: elastic stable intramedullary nailing (ESIN); Hybrid: ESIN for the radius and plate–screw fixation for the ulna; HIT: titanium intramedullary rod; RESIN: resorbable intramedullary rod; HF: hybrid fixation; I-KW: intramedullary Kirschner wire.

The studies investigated different treatment methods, including plate osteosynthesis (PO), Titanium Elastic Nails (TEN rods), a combination of TEN rods and Kirschner wires (TENK), open reduction and internal fixation with plate and screws (ORIF), plate–screw fixation (FPP), hybrid fixation (FH), conservative treatment with splint and cast (CO), double-plate fixation (FPD), intramedullary fixation with Kirschner wires (FK), and Epibloc system fixation (FES). Follow-up duration varied across studies, providing a comprehensive view of the effectiveness and complications associated with each treatment method. Complications observed in the included studies are summarized in Table 2.

**Table 2:** Summary of complications/adverse events of the treatments reported in the evaluated studies.

Author/Year	Complications/Adverse Events
Barua et al.^16^	Infections, transient neuropraxia.
Duran Topak et al.^17^	Surgical site infection, refracture, pin entry irritation, hypertrophic scar.
Freese et al.^18^	Wound dehiscence, superficial infection, difficulty removing ulnar rod, finger flexion contracture, transient neuropraxia, implant migration.
Zeybek et al.^19^	Superficial infection, soft-tissue irritation, pseudoarthrosis, delayed union.
Soudy et al.^20^	Superficial infection, superficial radial nerve injury, residual nonunion of the radius.
Shihora et al.^21^	Elbow stiffness, hypertrophic scar, superficial infection, malunion.
Zhu et al.^22^	Refracture, nonunion of the radius, superficial infection.
Di Giacinto et al.^23^	Refracture, malunion, nonunion, superficial infection.
Jain et al.^24^	Superficial infection, nonunion, delayed union, refracture.
Pogorelić et al.^25^	Skin irritation, refracture, pseudoarthrosis.
De Vitis et al.^26^	Skin irritation.
Wu et al.^27^	NR (not reported).
Acharya et al.^28^	Skin irritation over prominent ulnar nail, superficial infection at nail entry site, ulnar nail backout.
Zheng et al.^29^	Superficial infection, superficial radial nerve palsy, soft-tissue irritation, refracture, nonunion.
Dávid et al.^30^	Re-displacement, irritation, skin perforation, superficial radial nerve injury.
Guzel et al.^31^	Superficial infection, soft-tissue irritation, refracture, pseudoarthrosis.

Among the most frequent complications, we observed superficial infections. Other common events included refractures, transient neuropraxia, skin irritation, and hypertrophic scarring, underscoring the variability in patient responses to different treatment methods. More severe complications—such as pseudoarthrosis, malunion, and nonunion—were also reported.

Regarding methodological quality, all studies assessed with the MINORS Scale were rated as good,^16-31^ each scoring 11 points or higher in the overall assessment, as shown in Table 3.

**Table 3:** MINORS Scale: 0 (not reported), 1 (reported but inadequate), or 2 (reported and adequate). The quality of each included study was defined based on the total score as poor (<5), fair (6–10), or good (≥11).

Study	Clearly stated aim (a)	Inclusion of consecutive patients (b)	Prospective data collection (c)	Appropriate endpoints (d)	Unbiased assessment of study endpoint (e)	Adequate follow-up period (f)	Loss to follow-up <5% (g)	Prospective calculation of study size (h)	Total score	Study quality
Barua et al.^16^	2	2 / n = 40	1	2	0	2	2	0	11	Good
Duran Topak et al.^17^	2	2 / n = 52	1	2	0	2	2	0	11	Good
Freese et al.^18^	2	2 / n = 102	1	2	0	2	2	0	11	Good
Zeybek, Akti et al.^19^	2	2 / n = 51	1	2	0	2	2	0	11	Good
Soudy et al.^20^	2	2 / n = 18	2	2	0	2	2	0	12	Good
Shihora et al.^21^	2	2 / n = 73	2	2	0	2	2	0	12	Good
Zhu et al.^22^	2	2 / n = 56	2	2	0	2	2	0	12	Good
Di Giacinto et al.^23^	2	2 / n = 43	1	2	0	2	2	0	11	Good
Jain et al.^24^	2	2 / n = 65	1	2	0	2	2	0	11	Good
Pogorelić et al.^25^	2	2 / n = 173	1	2	0	2	2	0	11	Good
De Vitis et al.^26^	2	2 / n = 44	1	2	0	2	2	0	11	Good
Wu et al.^27^	2	2 / n = 26	2	2	0	2	2	0	12	Good
Acharya et al.^28^	2	2 / n = 31	1	2	0	2	2	0	11	Good
Zheng et al.^29^	2	2 / n = 137	1	2	0	2	2	0	11	Good
Dávid et al.^30^	2	2 / n = 86	1	2	0	2	2	0	11	Good
Guzel et al.^31^	2	2 / n = 78	1	2	0	2	2	0	11	Good

Given this scenario, the meta-analyses focused specifically on changes in the following clinical parameters:

- Adverse events: The analysis of adverse events, based on eleven included studies^16-31^ comparing the risk of adverse events between the experimental group (TEN rods and/or Kirschner wires) and the control group (other treatments), indicated a statistically significant increase in risk in the experimental group, with a risk ratio (RR) of 1.35 (95% CI: 1.03 to 1.76). This suggests that participants in the experimental group had a 35% higher risk of experiencing adverse events than those in the control group. Moreover, there was no evidence of significant heterogeneity across studies (I² = 0%, p = 0.54), indicating consistent results among the studies. (Figure 2)
- Surgical time: Pooling surgical time reported by eight studies^16,17,19,22,23,27,29^ that compared operative duration between the experimental and control groups showed a combined mean difference (raw mean) of −12.42 minutes (95% CI: −13.75 to −11.10) under the fixed-effect model, indicating a significant reduction in surgical time for the experimental group. Under the random-effects model, the mean difference was −21.62 minutes (95% CI: −33.30 to −9.94), likewise indicating a significant reduction but with greater between-study variability. In addition, heterogeneity was high (I² = 95%, p < 0.01), indicating substantial variability across individual study results. (Figure 3)
  These results suggest that, on average, the TEN-rod and/or Kirschner-wire group showed a significant reduction in surgical time compared with other treatments. However, the high heterogeneity among the studies indicates that these results may vary substantially depending on the specific characteristics of each study, such as differences in surgical protocols, surgeon experience, or patient-related variables.
- Length of Stay: The meta-analysis pooled the results of three studies^16,17,27^ that compared hospital length of stay between the experimental and control groups and showed that the combined mean difference between the experimental and control groups was −1.79 days (95% CI: −2.20 to −1.37) in the fixed-effect model, suggesting a significant reduction in length of stay for the experimental group compared with the control.
  Furthermore, heterogeneity among the studies was high, with I² = 93% (p < 0.01), indicating substantial variability across study results. Overall, the findings indicate that, on average, the experimental group experienced a significant reduction in hospital length of stay compared with the control group. However, the high heterogeneity suggests that the effects may vary significantly between studies, which may be related to differences in clinical context, interventions performed, or patient characteristics.
  In the additional analysis performed to evaluate hospital length of stay regardless of the control group, five studies^16,17,24,25,27^ comprising a total of 307 patients were included. The common-effect model indicated a combined mean of 3.46 days (95% CI: 3.36 to 3.56), suggesting a similar average length of stay across the included studies. Heterogeneity was high, with an I² of 100% (p < 0.01), indicating substantial variability among the studies. The τ² value of 2.9467 reflects this high heterogeneity, possibly due to differences in inclusion criteria, interventions, or study populations.
- Fracture Consolidation (union) Time: The analysis included six studies^17-19,22,29,31^ that compared the experimental and control groups regarding fracture union time. The fixed-effect model indicated a raw mean difference of 0.99 (95% CI: 0.61 to 1.36), suggesting that the experimental group had a longer recovery/consolidation time compared with the control group, with a statistically significant effect. Moreover, heterogeneity was moderate to high, with an I² of 73% (p < 0.01), indicating substantial variability among the included studies.

These findings suggest that the experimental group, on average, had a longer consolidation time than the control group, particularly when the fixed-effect model is considered. However, the random-effect model, coupled with high heterogeneity, demonstrates considerable uncertainty, meaning that results may vary substantially across studies. The interpretation of these findings should therefore take into account these variations and the possibility that the observed effects may not be consistent across different clinical settings.

## DISCUSSION

A comprehensive analysis of the literature indicated that most diaphyseal forearm fractures in children can be managed non-surgically through cast immobilization, a method that has shown excellent outcomes.^32,33^ However, fractures not eligible for conservative treatment generally require surgical intervention. Despite the strong theoretical basis supporting these concepts, there is still no global consensus on the best treatment strategy, particularly for unstable fractures, where surgical fixation is considered indispensable.^6^ From this perspective, the aim of this systematic review was to compare the clinical outcomes of children with forearm fractures who underwent surgical treatment with intramedullary fixation using TEN rods and Kirschner wires.

The analysis of the included studies revealed significant variability in clinical outcomes among the different treatment methods. Some studies highlighted the advantages of TEN, reporting a lower complication rate and faster recovery, while others suggested that K-wires might provide greater stability for certain types of fractures. These discrepancies underscore the need for a careful evaluation of treatment systems, considering the individual characteristics of each patient and the nature of the fracture.

Our data showed a predominance of male participants, a finding consistent across all studies, reflecting a higher incidence of trauma in this population. Furthermore, the mean age of participants ranged from 8.32 to 14.2 years, indicating a wide age distribution within the study groups and, therefore, relevant diversity in treatment responses depending on age.

The evaluated studies demonstrated differences in preferences and treatment outcomes. For instance, Barua et al.^16^ reported that TEN fixation significantly reduced surgical time compared to plate osteosynthesis. Similarly, Duran Topak et al.^17^ corroborated these findings, observing that TEN rods provided a shorter fracture consolidation time, although no significant differences were noted in functional outcomes or complication rates between TEN and PO. Soudy et al.^20^ also emphasized that TEN is safe and effective for forearm fractures, with most patients achieving good functional results. Likewise, Wu et al.^27^ and Acharya et al.^28^ found that TEN offers advantages such as shorter operative time and reduced fluoroscopic exposure compared to the use of K-wires.

Despite the benefits of TEN, some comparative studies have reported divergent outcomes. Freese et al.^18^ found that intramedullary fixation (IMN), which includes the use of TEN, was associated with a significantly higher complication rate and greater need for reoperations compared to plate osteosynthesis (PO).

Hybrid fixation, which combines TEN rods with plate–screw fixation, demonstrated distinct advantages. Zeybek and Akti^19^ observed that hybrid fixation resulted in shorter incision length and reduced operative time compared to PO, while providing an effective combination of the benefits of both techniques. Similarly, Guzel et al.^31^ confirmed that this strategy offered a good balance between surgical duration, blood loss, and immobilization time.

The plate–screw fixation (PO) technique was investigated by De Vitis et al.,^26^ who found it to be safe and effective for the treatment of distal forearm fractures, providing superior functional outcomes with minimal need for postoperative rehabilitation compared to fixation with K-wires and casting.

Hybrid fixation, according to Zheng et al.,^29^ also demonstrated advantages over double-plate fixation, including shorter surgical times, reduced blood loss, and faster union rates for the ulna. However, Zhu et al.^22^ and Dávid et al.^30^ noted that hybrid fixation and resorbable intramedullary rod techniques, although effective and associated with lower complication rates, still require further studies to validate their long-term efficacy.

Shihora et al.^21^ reported that cast immobilization achieved a higher bone union rate compared to TEN fixation. However, TEN fixation was effective when conservative treatment alone was insufficient. In the study by Di Giacinto et al.^23^ although K-wire fixation demonstrated faster bone union, plate–screw fixation (PO) was associated with fewer complications. Regarding complication rates, Jain et al.^24^ reported an overall complication rate of 41.5% with TEN fixation, emphasizing that cases requiring open reduction showed more complications, despite most patients achieving good to excellent functional outcomes. According to Pogorelić et al.^25^ intramedullary fixation with titanium elastic rods demonstrated a relatively low complication rate, and most patients achieved complete radiographic healing within an average of 6.8 weeks.

Given this context, the choice of technique for treating double diaphyseal forearm fractures should weigh several factors, including patient age, fracture severity, and surgeon experience. TEN is effective, offering advantages in operative time and recovery, but it may be associated with higher complication rates and reoperation needs when compared with open reduction and internal fixation. Hybrid fixation appears to be a promising alternative, combining the benefits of TEN and plate–screw fixation (PO), with favorable operative characteristics. Further studies are needed to confirm the effectiveness of techniques such as resorbable intramedullary rods and hybrid fixation, particularly with respect to long-term outcomes and complication rates.

Our analysis of complications revealed a wide range of adverse events associated with different surgical treatments for pediatric forearm fractures. Among the most frequently reported, superficial infections stand out as a recurrent issue—cited by Duran Topak et al.^17^ Freese et al.^18^ Soudy et al.^20^ and others. Although treatable, such infections can prolong recovery and increase patient discomfort, often requiring additional interventions.

Beyond infections, refractures and transient neurapraxia were also commonly observed. Refractures were reported by Duran Topak et al.^17^ Di Giacinto et al.^23^ Guzel et al.^31^ e Zhu et al.^22^ whereas transient neurapraxia was documented by Barua et al.^16^ and Freese et al.^18^ Even when transient, neurapraxia can impact limb function and warrants continuous monitoring.

Studies by Zeybek e Akti^19^ and Pogorelić et al.^25^ highlighted occurrences of pseudarthrosis and malunion—serious complications that impair bone consolidation and may necessitate further surgery. Pseudarthrosis, in particular, is worrisome because it signals failed bone healing, prolongs recovery, and can require additional treatment.

There was agreement across several studies regarding the prevalence of these complications; however, some discrepancies emerged. While Freese et al.^18^ reported issues such as difficult removal of the ulnar rod and implant migration, these were not mentioned by other authors, suggesting that such problems may be linked to technical particulars or surgeon experience. In addition, Acharya et al.^28^ described a more specific complication—skin irritation over a prominent ulnar nail—not reported elsewhere, possibly reflecting differences in surgical technique or approach.

Taken together, these variations underscore the need to individualize treatment and ensure rigorous postoperative follow-up to prevent and manage adverse events effectively. The choice of surgical method should consider not only fracture management efficacy but also each technique's complication profile, with the goal of minimizing risks and optimizing patient recovery.

## CONCLUSIONS

Trial quality, assessed with the MINORS tool, was rated good. The meta-analysis for adverse events showed a statistically significant increase in risk in the experimental group compared with controls, indicating that participants in the experimental group were more likely to experience adverse events; no significant heterogeneity was identified across studies.

Regarding operative time, the pooled (raw) mean difference between the experimental and control groups was −12.42 minutes under the fixed-effect model, indicating a significant reduction in surgical time for the experimental group. Under the random-effects model, the mean difference was −21.62 minutes, likewise indicating a significant reduction but with greater between-study variability. For length of stay, there was a significant reduction favoring the experimental group versus controls.

The meta-analysis also showed that, for fracture consolidation, the experimental group had a longer recovery/consolidation time than the control group, with a statistically significant effect. However, the random-effects analysis, together with high heterogeneity, indicates considerable uncertainty—i.e., results may vary markedly across studies. Interpretation should therefore account for this variability and the possibility that effects are not consistent across clinical settings.

Conservative cast treatment is broadly effective for stable, simple fractures, yielding excellent outcomes with relatively low complication rates. In unstable or complex fractures, however, surgery becomes indispensable.

Among surgical techniques, elastic intramedullary fixation with TEN rods offers important advantages—faster recovery and shorter operative time—and is particularly effective when conservative treatment fails. Nonetheless, TEN is associated with complications such as reoperations and refractures, and with higher overall complication rates compared with plate-and-screw osteosynthesis. Kirschner wires (K-wires) can achieve rapid union, but they carry higher risks of complications, including infections and transient neurapraxia.

Accordingly, the choice between surgical and conservative management should be individualized based on fracture characteristics, patient age, and surgeon experience, carefully weighing each method's risk–benefit and complication profile. Further long-term studies are needed to confirm functional outcomes and complication patterns, especially for emerging techniques such as hybrid fixation and resorbable intramedullary rods.
